# Extracts from the Minutes of the New York Pathological Society

**Published:** 1855-05

**Authors:** 


### ECLECTIC DEPARTMENT,

### AND SPIRIT OF THE MEDICAL PERIODICAL PRESS.

---

### *Extracts from the Minutes of the New York Pathological Society.*

*Dec.* 27, 1854. Dr. Clark presented a specimen of blood, from a patient who died of gangrene of the feet, nose, fingers, etc. The following history has been furnished with the case:

"L. W., aged 28 years, was admitted to the hospital about three weeks since, with a sore mouth, which she attributed to some pills that were administered by a physician; she also complained of great tenderness of her feet, which were blue and cold for about three inches above the ankle. Her nose and two fingers of the left hand were in the same condition. Afterward, the other hand and the ears became affected, and, still later, other parts. She died the night before last. At the autopsy, all the internal organs were found healthy. The iliac, femoral, radial, tibial, and brachial arteries were examined, and found pervious and healthy."

The coloring matter seemed to be diffused through the whole mass of the blood, and not to be confined to the red corpuscles, which were very few and strong. The lymph corpuscles were also few, but large.

Dr. Clark exhibited a specimen of cancer of the cardia, of interest from the valvular form of the growth obstructing the passage of the food.

Dr. Peaslee thought this case very interesting, from the satisfactory manner in which the *post-mortem* appearances explained the *pre-mortem* symptoms. Some of the latter, however, reminded him of a case of his, in which malignant disease was entirely out of the question. The patient was a medical pupil, 26 years of age, and perfectly well in every other respect, who for three or four years previously could not pass any solid food into his stomach by ordinary acts of deglutition. It would apparently accumulate in the œsophagus until six or eight ounces had thus been collected, when a sensation of great tension and distress would at once occur, and which would be promptly followed by the ejection of the food, unless great quantities of water, two or three tumblers full, were swallowed with the greatest rapidity. In this way the food would be carried at once into the stomach, and instantaneous relief afforded. The difficulty had not increased at the time alluded to for the last two years, nor did it increase in the least degree for four years subsequently. He is now a foreign missionary. Dr. Peaslee supposed that there must have been a dilatation of the œsophagus, which might have been produced by some valvular development of the cardia, though certainly not from malignant disease.

Dr. W. Parker inquired if the œsophageal bougie had been used by way of exploration.

Dr. Peaslee said that it had been by others before he saw the case, but without detecting dilatation.

Dr. Parker asked if pouches in the œsophagus were common on record.

Dr. Peaslee said that his attention had been directed to that question at the time, but that he could not find any case of the kind.

Dr. Post had seen a case of disease of the œsophagus which was thought to be malignant, but which proved to be chronic abscess, as it afterward burst and discharged itself, having existed several months.

Dr. Clark remarked that one difference between his case and that of Dr. Peaslee's was, that the patient in the former was diabetic, and in the latter generally healthy.

Dr. Clark presented a lung in which gangrene had taken place. The disease had gone on, and successfully passed the crisis, when the patient died suddenly from pulmonary hæmorrhage. A large cavity was found in the lung, lined by a pyogenic membrane. Dr. C. remarked that they were able to assert the precise time the membrane was formed and commenced secreting pus.

Dr. Batchelder asked how large a proportion of those affected with gangrene of the lung recovered.

Dr. Clark thought from two-fifths to one-half.

Dr. Batchelder gave an account of a patient he had some thirty years since, affected similarly to Dr. Clark's patient. His patient also died from pulmonary hæmorrhage.

Dr. Clark thought that, in his case, gangrene was not preceded by inflammation of the lung. He also felt quite sure that this is ever the case, and that the gangrene is the existing cause of the accompanying pneumonia.

Dr. W. Parker thought that this was contrary to the generally received opinion.

Dr. Clark did not feel satisfied as to the reason. He had investigated the subject with Dr. Swett, and, like Dr. Creswell, found the result as he had just stated. Dr. C. remarked that there were other tissues that never became gangrenous in consequence of inflammation.

Dr. Batchelder thought Dr. Clark's opinion was correct, and that the inflammation was an effort at reparation.

Dr. Clark thought the inflammation a result of irritation.

Dr. Peaslee would receive the proposition that inflammation of the lung never produces gangrene of its substance with much hesitation. Certainly, analogy pointed in the opposite direction. Inflammation is admitted to produce gangrene of the areolar tissue and of the brain, and he could see no impossibility or improbability in the idea that it might produce similar effects and consequent sloughing in other parts or organs well supplied with blood. Moreover he had, within the past month, seen a case of gángrene of the lung in which all the usual signs of pneumonia had been present for two and a half weeks, as nearly as he could judge from the facts of the case, as detailed at the consultation visit, before any signs of gangrene manifested themselves. He had also arrived at a similar conclusion in another instance, though aware of Dr. Carswell's opinion on that subject. Though, therefore, he believed that gangrene of the lung is very often, and perhaps generally, produced by other causes than inflammation, he could not accede to the proposition that inflammation of the lung never produces it.

Dr. Clark thought there was no disputing the position that serous membrane never mortifies as a direct consequence of inflammation. On the other hand, if cerebral substance becomes inflamed, gangrene or softening is very apt to follow. Next to the cerebral substance, gangrene is apt to follow inflammation in the areolar tissue. The next tissue in order is the mucous membrane and the skin, when the tissues below are dead; but not often otherwise. Dr. C. thought the membrane lining the air-vesicles more nearly allied to mucous than to serous membrane.

Dr. Peaslee still thought the analogy held good.

Dr. Clark remarked that analogies are good for nothing in a case like this, unless closely analyzed, and that the case spoken of by Dr. P. was too vague to be of any practical value, inasmuch as Dr. P. had not seen the case previously to consultation.

Dr. Peaslee replied that, in one sense, he entirely agreed with Dr. Clark as to the value of analogies in respect to the proposition under discussion. In another, however, he entirely disagreed with him. Of course, no such proposition can be established by analogies alone. Nothing but observation can establish any such positive statement. But analogies may be of great value in the way of putting us on our guard against too readily adopting any such exclusive proposition, and may even throw a doubt upon it, if they all point the other way; and it was on both these accounts that he had suggested them. In regard to the vagueness of the case to which he had alluded, he would say that he had not intended to give an account to the society other than vague, since he only alluded to it rapidly, as a case inducing him to doubt the correctness of the proposition, that inflammation never produces gangrene of the lung.

Dr. Clark further remarked that the branches of the pulmonary artery anastomose only with the return vessels. Hence, if a trunk going to any given amount of the lung be obstructed, that portion of the lung must die.

Dr. Peaslee replied that unless we assume also that inflammation cannot obstruct the minute vessels in the lung, as it was proved to do elsewhere, he should consider that the peculiarity of the circulation just alluded to by Dr. Clark would render the lungs even more liable to gangrene as a result of inflammation. For if the latter should chance to obstruct a single terminal artery entering a lobule, the whole lobule must necessarily die, and so in proportion to the size of the vessel occluded.

Dr. Clark thought that inflammation can affect only the capillaries, while in his case he found obstruction of the trunks going to the part by stricture or obliteration of the vessels.

Dr. Weber presented the entire osseous system of a woman who died of cancer in Amsterdam.

Dr. Clark thought this the most extensive case of carcinoma ever presented to the society. It also confirmed the opinion of Dr. Markoe upon a case presented to the society, some two years since, of a mammary cancer, which seemed to retrograde, leaving the breast finally very hard, yet afterward manifesting itself in the peritoneum.

Dr. Post remarked upon the great number of foci in Dr. Weber's case without any great centre.

Dr. Parker mentioned the case of a female, who resided upon Staten Island, whose breast had been diseased, and remained hard thirty years, who finally died with cancer of the uterus.

Dr. Weber also presented an amputated leg, the history of which was given by Dr. Parker, who had seen the case a few days previously in consultation. Dr. P. found that the patient had been troubled with a pain in the foot since last August, that he had also been troubled with a small tumor in the popliteal space. This tumor had been poulticed for a considerable time by the then attending physician, who, under the impression that it was an abscess, had finally laid it open, but was disappointed in finding nothing but blood. Dr. P. could not detect pulsation, and felt inclined to look upon it as a malignant growth, and, with this impression, advised amputation. Dr. Weber saw the case the next day, and, after consultation with Dr. Parker, amputated at the lower third of the thigh. Upon dissecting the tumor, it was found to be an aneurism of the popliteal artery.

Dr. Weber remarked that the bone was also somewhat diseased, and that the amputation was justifiable. He added that the patient had been troubled with stiffness of the knee-joint nearly a year, with considerable œdema of the lower extremity, but only for the past three weeks had the tumor increased rapidly. This rapid increase, Dr. W. considered to be owing to rupture of the aneurismal sac.

Dr. O'Rorke presented a specimen of cancer of the pancreas, taken from a young man twenty-four years of age, who had always been perfectly well until last spring. When first called to him, Dr. O'R. distinctly heard the bellows murmur over the precordial region, which decreased gradually until the fourth week, when it entirely disappeared. The tumor was mostly situated upon the left side. Dr. Metcalfe saw the patient in July, but felt quite uncertain as to the nature of the disease. Another physician saw him in September, and thought he could cure him. He first bled him in the recumbent posture *ad deliquium*. He then gave him a powerful emetic, followed by mercury to salivation. At this stage of the treatment Dr. O'Rorke saw him, and continued to visit him until December 24, when he died.

Dr. Metcalfe remarked that this was a case of unusual interest, on account of the very many adhesions with surrounding organs, and that he did not recollect the record of any case where there was so much destruction of the walls of the stomach. He found, on microscopical examination of the specimen, minute elongated cells, a very large quantity of oil globules, and cells with large nuclei.

Dr. Clark asked if the dissection had been so made that it was sure that he pancreas was really the seat of the disease? This organ, he thought, was very seldom the seat of cancerous disease, but that cancer often manifested itself in the areolar tissue, displacing organs which, of themselves, are free from disease.

Dr. Batchelder remarked that he had repeatedly observed pulsation in abdominal tumors to disappear. He asked the cause. Thought such tumors were generally malignant.

January 10, 1855.

Dr. Clark presented a specimen of parasitic vegetable growth, removed by Dr. Kissam from the ear of a lady æt. sixty years. The ear had been syringed after emollient applications, for the removal of a suspected accumulation of inspissated cerumen, causing deafness. The edge of a white membrane presenting, Dr. Kissam was able to withdraw by forceps what seemed to be a white membranous lining of the whole lower external ear. It was moulded to its walls, having on its surface an impression of the bones of the tympanum. On its removal, the hearing on that side was very much improved. The other ear was to receive the same treatment. On microscopic examination, the mass of the membranous accretion was found to consist of a vegetable product, belonging to the same general class as the *bidium albicous* described by Dr. Clark at the meeting of this society January 25, 1854, but unlike it in being unicellular, or composed of cells of unusual length. The stem had somewhat branched, and the spores appeared to be more or less vested in the epithelial cells. Dr. Clark thought that this plant probably grew from the epithelial lining of the external ear. He believed that a vegetable growth in the ear had not before been recognized as a cause of deafness.

Dr. Clark also presented a mass of fibrous tumors, weighing several pounds, springing from the fundus and body of a uterus. The woman from whom the specimen was taken, died suddenly, and the partial history of the case obtained was of no value.

Dr. Dalton showed a malformation of the heart, from a mulatto male infant, in which the organ had but two cavities, viz: one auricle and one ventricle. The case occurred in the practice of Dr. H. W. Brown. The infant was the fifth child of its mother, but the first by a new husband, the mother having married the second time. All the other children were perfect except the first, which was born before its time, and lived but a few hours. The infant from whom the specimen was taken, was born at full time, and had no other malformation. It appeared quite well for eighteen hours after birth, except for some coolness of the skin. It then began to show signs of distress, which were attributed to flatus, grew constantly cooler at the surface and more dark colored about the face, and died, without any other remarkable symptom, about twenty-four hours after birth. The heart was of natural size and nearly of natural shape externally, but, on being opened, was found to consist only of the right auricle and ventricle, the left cavities being entirely wanting. The blood passed out of the right ventricle through the pulmonary artery, which sent two small branches to the lungs, and then continued onward, by the ductus arteriosus as a large trunk, to the arch of the aorta. Part of the blood then passed down through the thoracic aorta, and part returned, in a backward direction, through the aortic arch, which, after supplying the head and upper extremities, returned to the heart, and terminated in the coronary arteries.

Dr. Dalton also exhibited a horny epidermic growth from the forehead of a man about twenty-five years of age, which had been removed by Dr. Detmold. The horny protuberance was a quarter of an inch long, of a brownish color, and fissured longitudinally. Its base was seated in an enlarged cutaneous follicle.

Dr. Sayre exhibited a portion of the walls of the abdomen of a patient into whose peritoneal cavity he had introduced a lead seton, for the purpose of producing adhesion of the walls of the cavity, in a case of ascites dependent on organic disease of the heart. Also, a portion of the intestines which were agglutinated by recent lymph. The most serious symptoms caused by the accumulation were relieved by the operation, but the patient died with diarrhœa. The autopsy showed that general peritonitis, with adhesion, had been excited by the seton, but that there had not been a sufficiently free exit to accumulating fluid. Dr. Sayre thought that it would have been better to have left in a canula, as in Dr. Hart's recent operation. Dr. Sayre had operated once before, under similar circumstances. The man was greatly oppressed by accumulation of serum from organic disease of the heart. Having drawn off twelve quarts of fluid, he introduced the lead seton, and made incisions over the malleoli. In twelve days, sixteen quarts of serum escaped by these incisions. Peritonitis having been excited in a mild degree by the seton, no reäccumulation in the abdomen took place, and the patient got about, and is now living in moderate health. A history of the first case was furnished for the archives of the society.

Dr. Clark inquired as to the result of puncture of the abdomen for the removal of serous fluid, when the wound has not been closed? His experience had been that the operation led to unpleasant results in a large proportion of cases. Dr. Clark thought that the fact, that the friction of the bowels was apt to be impaired when general agglutination took place, was an objection to the introduction of a foreign body like the seton, which would excite adhesive inflammation.

Dr. Sayre considered this result of the operation the less of two evils. In answer to Dr. Batchelder, he stated that the bowels of the first patient mentioned, were regular for ten days after the operation, until diuretics were administered, when diarrhœa set in.

Dr. Conant suggested the whole sac of the vagina, when the peritoneum is reflected from the rectum, as a suitable point for puncture of the abdomen in cases of accumulation of fluid.

Dr. Sayre thought that the wound would not generally heal. He inquired the result of operations for ascites by injection of the abdomen.

Dr. Cochran alluded to a case reported by Dr. Otto Rotton, of a case of ascites of twenty years' standing cured by the injection of tr. iodine.

Dr. Emmet exhibited a heart, with the following history:

A female, a native of Ireland, æt. 30 years, was admitted to the Emigrant Hospital, Ward's Island, Nov. 30, 1854. On admission, the face, body, and lower extremities were very œdematous, with effusion into the peritoneal sac. The left side of the chest was dull on percussion as high as the spine of the scapula, at which point the respiratory murmur could be heard, although very feeble. The resonance on the right side was nearly normal. The lung was somewhat œdematous. There existed an extensive effusion into the pericardium. The right ventricle was hypertrophied and dilated with tricuspid regurgitation. Heart's impulse very feeble, with a double murmur over the apex. Pulse 120 per minute, small and feeble. She died Dec. 28, 1854.

*Autopsy, forty-eight hours post-mortem.* The effusion was very great in the left pleural cavity, with the lung compressed to nearly half its normal size, and firmly bound down on its posterior surface with a dense band of fibrin about an inch wide, extending from apex to base. The pericardium contained about a pint of fluid. The right ventricle was dilated. The mitral valves were glued back to the side of the ventricle so as to form a complete tube. Other organs healthy.

Dr. Isaacs related an instance of enchondroma in a negro, connected with the knee-joint. A tumor was noticed in the popliteal space half the size of a hen's egg. The popliteal artery and vein were found to be stretched over it. It was enchondromatous in its structure, and was attached to the posterior ligament of the tibia. There were several smaller masses disengaged, from the size of a shot to that of a pea, and another, very firm, connected not only with the cartilage of the tibia, but even of one substance with the joint itself.

Dr. Sayre thought these growths to be probably the result of inflammation of the joint, as in two instances where he had opened joints where ulcerative disease existed, the loss of substance was repaired by material similar to this, so far as could be determined by the eye and probe.

Dr. Finnell exhibited three atrophied infants, and stated that similar cases came under the notice of the coroners frequently. They are found only in the poorer districts of the city, are fed on tea, coffee, and bad milk, are teased with vermifuge medicines, and generally die in convulsions. On examination after death, the head is found enlarged, weighing generally as much as the body, with considerable serous effusion in the ventricles. The children are under six months old; there is no sign of disease but atrophy. No fat is found except in the orbits and under the malar bones. At one year of age there is enlargement of the cervical glands, and after one year generally tubercles. Dr. Finnell said that he considered the effusion into the ventricles as anemic, and not as inflammatory effusion. The parents were generally healthy.

Dr. Finnell exhibited a specimen of internal strangulation of the small intestine. The patient, a man æt. 27, was well until forty hours before death, when he was attacked with cramp and pain in the bowels. Then he took peppermint and brandy and went to bed. The same night he took castor oil, but with no relief. Other cathartics were administered, but were vomited. Five hours before death he spoke of feeling better. At the autopsy, about two feet of the ileum was passed under the sigmoid flexure of the colon, where the peritoneum was deficient at the upper part of the rectum, and there twisted on itself.

Dr. Batchelder thought that the stethoscope would assist in the diagnosis of internal strangulation of the bowels. He is able to trace peristaltic motion through the whole course of the bowels, can hear the contents of the bowels drop through the ileocolic valve, and can detect obstruction of the bowels.—*N. J. Med. Reporter.*

